# Management of Colorectal Cancer with Synchronous Liver Metastases: A systematic review of national and International Clinical Guidelines (CoSMIC-G)

**DOI:** 10.1016/j.sopen.2024.10.009

**Published:** 2024-10-31

**Authors:** Anthony K.C. Chan, Ajith K. Siriwardena

**Affiliations:** Regional Hepato-Pancreato-Biliary Surgery Unit, Manchester Royal Infirmary, Manchester, UK; Faculty of Biology, Medicine and Health, University of Manchester, Manchester, UK

**Keywords:** Colorectal cancer, Synchronous liver metastases, Guidelines

## Abstract

**Introduction:**

The contemporary management of patients with colorectal cancer and synchronous liver metastases is complex. This study appraises the recommendations made by national/international guidelines for the diagnosis and management of patients with synchronous liver metastases from colorectal cancer.

**Methods:**

A systematic review of national and international guidelines published between 2011 and 2024 was carried out using PubMed, OvidSP and Guidelines International Network databases. The quality of guidelines was evaluated using the Appraisal of Guidelines for Research & Evaluation II (AGREE II) instrument. Guidelines were assessed for the quality of advice for specific scenarios. The protocol was registered with PROSPERO (CRD42021243744).

**Results:**

The search strategy returned ninety unique articles with 11 guidelines eligible for inclusion. Of these, one (9 %) guideline defined ‘synchronous disease’ at outset, eight (73 %) recommended neoadjuvant chemotherapy as first intervention. Seven (64 %) guidelines supported synchronous hepatic resection with colectomy. One (9 %) recommended against synchronous surgery.

**Conclusions:**

This study demonstrates important variations between international clinical guidelines on diagnostic workup and management of synchronous liver metastases in colorectal cancer. [167 words].

## Graphical abstract

**Figure:**
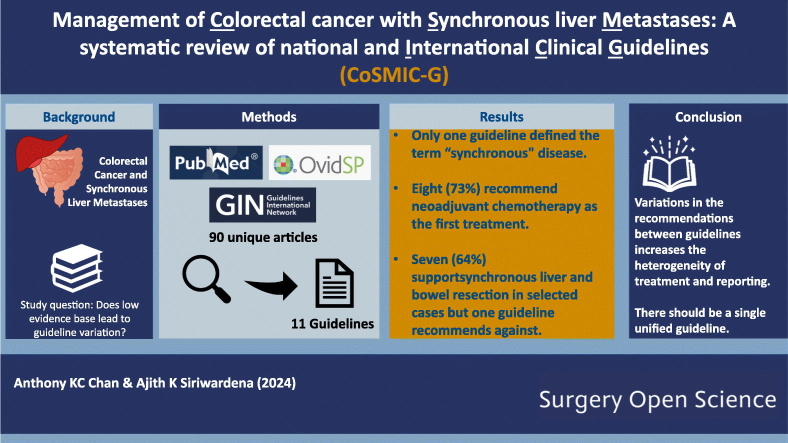

## Introduction

Contemporary management of patients with colorectal cancer and synchronous liver metastases is complex [1,2]. For patients of good performance status with hepatic metastases which are amenable to resection, the goal of treatment is to combine systemic chemotherapy (including radiotherapy for selected patients with rectal tumours) with removal of the macroscopic disease burden from both the primary site and the liver [3,4]. The role of chemotherapy (with or without biologic agents) for patients with limited metachronous liver metastases has been evaluated in two large, randomized trials [5,6]. In contrast, there is little high-level evidence to support the treatment options available in the synchronous setting. As a result, clinical guidelines carry particular importance in terms of guidance for decision-making for patients with synchronous colorectal cancer and liver metastases.

In this regard, the World Health Organization's (WHO) Advisory Committee on Health Research recommends that research evidence should inform best clinical practice [7]. Yet it is difficult for guidelines for the management of patients with colorectal cancer and synchronous liver metastases to provide high-quality evidence-based recommendations in a setting where knowledge accrues predominantly from case cohort studies and retrospective analyses. This lack of high-level evidence may mean that different guidelines make recommendations that vary from each other which in turn can exacerbate variation in practice.

Despite these potential limitations, guidelines carry importance in clinical practice as reference documents. Thus, the aim of this study is to undertake a systematic review of national and international guidelines for the management of patients with colorectal cancer with a specific focus on the advice for patients with synchronous liver metastases. This study focuses on using appropriate methodology to assess the quality of the guidelines and on the practical advice provided for diagnosis and management including operative treatment, the role of synchronous versus staged surgery and recommendations for integration of chemo(radio)therapy into treatment pathways.

## Methods

### Design

This is a systematic review of clinical guidelines with a specific focus on the management of patients with colorectal cancer and synchronous liver metastases. This review is reported in compliance with the Preferred Reporting Items for Systematic Reviews and Meta-Analyses (PRISMA) checklist [8]. The study protocol was registered with the PROSPERO register of systematic reviews (registration number CRD42021243744) [9].

### Eligibility criteria

Studies are included if they are national or international guidelines on the management of patients with colorectal cancer. This study has a specific focus on the management of the cohort with synchronous liver metastases. Regional guidelines within a country already covered by a national guidance document were excluded for the purposes of this study. The study period is the thirteen-years from January 2011 to January 2024. Where multiple versions or updates of guidelines exist, only the most recent version was included. The study was restricted to guidelines published in the English Language and in the case of guidelines originally published in languages other than English, these were only included if there was a publicly accessible English language translation. Systematic reviews and meta-analyses were excluded.

### Information sources

The National Library of Medicine PubMed and the Ovid SP® databases were queried for metastatic colorectal cancer guidelines using the keywords “colorectal cancer”, “liver” and “guidelines”. The guideline repository Guidelines International Network as well as colorectal and hepato-pancreato-biliary (HPB) specialist society websites were also searched for guidelines using the same search terms.

### Assessment of quality of guidelines

The Appraisal of Guidelines for Research and Evaluation II (AGREE-II) Instrument was used to appraise the quality of guidelines [10]. The AGREE-II Instrument is a 23-item framework which assesses the quality of healthcare guidelines over six domains. These domains cover the scope and purpose of the guidelines, stakeholder involvement, rigour of development, clarity of presentation, applicability, and editorial independence. Each item in the framework is scored between 1 (strongly agree) and 7 (strongly disagree), and a score for each domain is calculated using the assessment system described in the AGREE-II manual [10].

### Guideline advice on the diagnosis, staging and management of patients with colorectal cancer and synchronous liver metastases

Information was sought in relation to the advice given on selected aspects of the practical management of patients with colorectal cancer and synchronous liver metastases. These follow key steps of the treatment pathway but are not intended to cover all potential components at every stage. The first category addresses pre-operative diagnosis and staging. Here, the study focused on guideline recommendations on the use of contrast-enhanced magnetic resonance (MR) scanning to assess liver metastases and the use of ^18^fluoro-deoxyglucose positron emission tomography. Rectal MR scanning was not included as it is considered standard of care for assessment of patients with rectal tumours [11,12].

Next, guideline recommendations on sequence of treatment for patients with an elective presentation of colorectal cancer with synchronous liver metastases was sought. Here, guidelines were interrogated for recommendations on the use of neoadjuvant chemotherapy for elective presentations of patients with M1a metastatic disease [13]. In terms of treatment sequencing, guideline recommendations on the use of surgery as a first approach (either staged or synchronous) in patients with non-urgent presentations of M1a disease was also sought. It was accepted that it would be possible for guidelines to support both approaches [[11], [12], [13]].

If guidelines recommended synchronous hepatic and colonic resectional surgery, information was sought on whether they defined hepatic and primary tumour criteria for selection of patients for this approach. Recommendations on the choice of either the laparoscopic route or open surgery were not assessed. Finally, the guidelines were interrogated for the advice given on a series of specific management scenarios. These included questions on synchronous pulmonary metastasectomy in patients with synchronous disease and whether guidelines recommended synchronous surgery for synchronous liver lesions which demonstrate a complete response to systemic chemotherapy.

### Presentation of guideline recommendations

The American College of Chest Physicians (CHEST) modification of the Grading of Recommendations, Assessment, Development and Evaluation (GRADE) system is used [14]. This uses a “traffic light” system of both the strength of recommendation and the quality of the evidence on which that recommendation was made. Thus, recommendations range from “strong” to “weak” and the quality of evidence used to inform specific guideline points was categorised as ‘high’ (individual randomized trial or systematic review or meta-analysis of randomized controlled trials), ‘moderate’ (individual cohort or systematic review of cohort studies) or ‘low’ (individual series or systematic review of case-control studies). Guideline recommendations were ‘green’ if there was a strong recommendation from high or moderate quality evidence or a weak recommendation from high quality evidence, ‘amber’ if there was a weak recommendation from moderate quality evidence and ‘red’ if the recommendation was made from low or very-low quality evidence. If the guideline recommended against a specific treatment such as synchronous resection, this was noted with a black outline to the red indicator.

## Results

### Search strategy and study retrieval

**Fig. 1:**
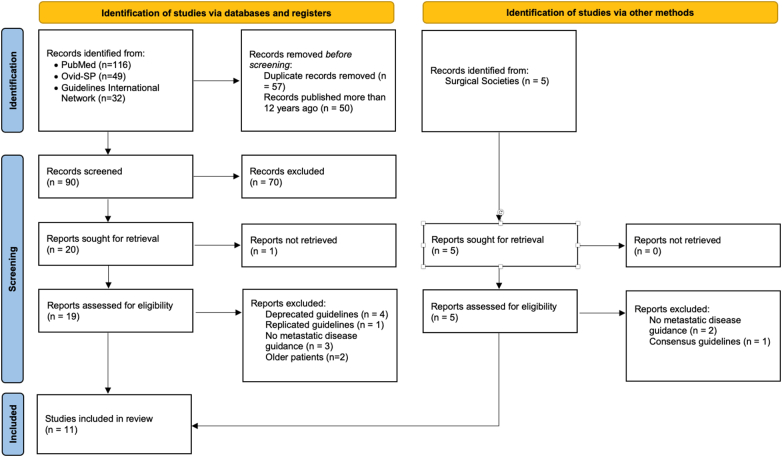
PRISMA Flowchart. Routes of identification of studies are demonstrated.

The search results are seen in the PRISMA flowchart (Fig. 1). The guideline search strategy returned ninety unique articles for screening, of which 19 colorectal cancer and metastatic colorectal cancer guidelines were identified and retrieved for assessment of eligibility. Ten of these were excluded as they were either overlapping guidelines or did not discuss the management of patients with liver metastatic disease. Two further reports were identified from the records of surgical society meetings. Thus 11 guidelines constitute the final study population for this review [12,13,[15], [16], [17], [18], [19], [20], [21], [22], [23]] (Table 1).

**Table 1:** Demographic of retrieved guidelines.

Year of publication	Abbreviation	Title	Body	Region	Level of development
2011	SFCD^15^	Management of patients with synchronous liver metastases of colorectal cancer. Clinical practice guidelines	SFCD; Association of Hepatobiliary Surgery and Liver Transplantation	France	National
2014	Saudi^16^	Saudi Oncology Society clinical management guideline series (colorectal cancer 2014)	Saudi Oncology Society	Saudi Arabia	National
2016	Brazil^17^	Brazilian Consensus on Multimodal Treatment of Colorectal Liver Metastases (Module 2: Approach to Resectable Metastases)	CB-IHPBA, SBCO, SBOC, CBCD, CBC, AHPBA	Brazil	National
2018	SEOM^18^	SEOM clinical guidelines for diagnosis and treatment of metastatic colorectal cancer	Sociedad Española de Oncología Médica	Spain	National
2018	CCA^19^	Clinical Practice Guidelines for the Prevention, Early Detection and Management of Colorectal Cancer	Australia Cancer Council	Australia	National
2019	JSCCR^20^	Japanese Society for Cancer of the Colon and Rectum guidelines 2019 for the treatment of colorectal cancer	Japanese Society for Cancer of the Colon and Rectum	Japan	National
2019	SNFGE^21^	Metastatic colorectal cancer: French intergroup clinical practice guidelines for diagnosis, treatments and follow-up	SNFGE, FFCD, GERCOR, UNICANCER, SFCD, SFED, SFRO, SFR	France	National
2021	NICE^12^	Colorectal cancer (NG151)	NICE	England	National
2023	CSCO^22^	Chinese Society of Clinical Oncology diagnosis and treatment guidelines for colorectal cancer (2023 update)	Chinese Society for Clinical Oncology	China	National
2023	ESMO^13^	Metastatic colorectal cancer: ESMO Clinical Practice Guideline for diagnosis, treatment and follow-up	European Society of Medical Oncology	Europe	International
2023	NCCN^23^	Colon Cancer	NCCN	USA	National

Abbreviations:

Société Française de Chirurgie Digestive (SFCD).

Capítulo Brasileiro da International Hepato-Pancreato- Biliary Association (CB-IHPBA); Sociedade Brasileira de Cirurgia Oncológica (SBCO); Sociedade Brasileira de Oncologia Clínica (SBOC); Colégio Brasileiro de Cirurgia Digestiva (CBCD); Colégio Brasileiro de Cirurgiões (CBC); Brazilian College of Surgeons (CBC); Americas Hepato- Pancreato-Biliary Association (AHPBA).

Sociedad Española de Oncología Médica (SEOM).

Cancer Council Australia (CCA).

Japanese Society for Cancer of the colon and rectum (JSCCR).

Société Nationale Française de Gastro-Entérologie (SNFGE); Fédération Francophone de Cancérologie Digestive (FFCD); Multidisciplinary Oncology Cooperative Group (GERCOR); Fédération Nationale des Centres de Lutte Contre le Cancer (UNICANCER); Société Française d'Endoscopie Digestive (SFED), Société Française de Radiothérapie Oncologique (SFRO); French Society of Radiology (SFR).

National Institute for Health and Care Excellence (NICE).

Chinese Society for Clinical Oncology (CSCO).

European society of Medical Oncology (ESMO).

National Comprehensive Cancer Network (NCCN).

### AGREE II scores (Table 2)

The standardised scores for each domain are shown in Table 2. The median score for scope and purpose of the guidelines was 92 % (range 22–100 %), for stakeholder involvement 50 % (6–83 %) and the median score for rigour of development was 58 % (4–93 %). The median score for clarity of presentation was 83 % (47–100 %). The median score for applicability was 44 % (8–85 %) and editorial independence 46 % (0–92 %) with five guidelines providing no information on either professional or financial conflicts of interest.

**Table 2:** Guideline standardised AGREE-II Scores.

Domain	1	2	3	4	5	6
	Scope and purpose	Stakeholder involvement	Rigour of development	Clarity of presentation	Applicability	Editorial independence
**SFCD 2011**	89 %	50 %	47 %	75 %	23 %	0 %
**Saudi 2014**	22 %	6 %	4 %	47 %	8 %	0 %
**Brazil 2016**	58 %	19 %	4 %	58 %	42 %	21 %
**SEOM 2018**	75 %	39 %	58 %	53 %	17 %	75 %
**CCA 2018**	97 %	67 %	76 %	92 %	46 %	88 %
**JSCCR 2019**	92 %	61 %	68 %	78 %	60 %	50 %
**SNFGE 2019**	67 %	47 %	49 %	83 %	29 %	4 %
**NICE 2021**	100 %	72 %	93 %	94 %	77 %	67 %
**CSCO 2023**	100 %	39 %	33 %	100 %	44 %	33 %
**ESMO 2023**	100 %	83 %	82 %	100 %	85 %	46 %
**NCCN 2024**	100 %	78 %	89 %	89 %	56 %	92 %

Guideline standardised AGREE-II scores.

### Guideline recommendations on aspects of clinical management (Table 3)

*i) Definition of synchronous colorectal cancer and liver metastases.*

**Table 3:**
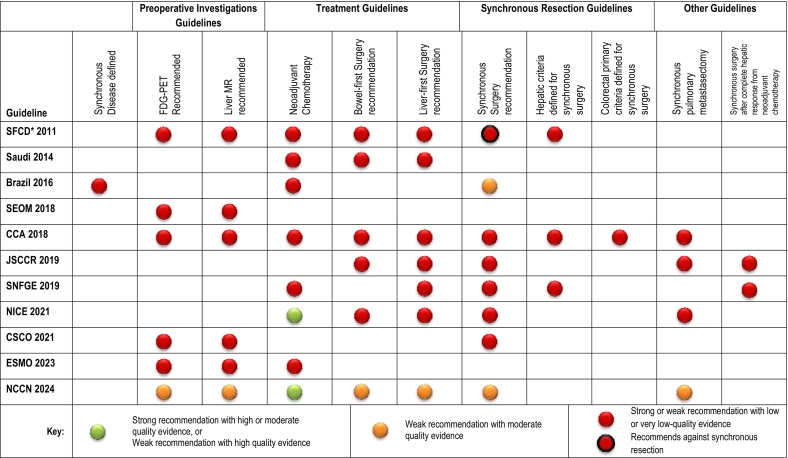
Guidelines on common clinical management questions at Tumour Board for metastatic colorectal cancer.

*Note that the SFCD guideline recommends against synchronous resection of liver and bowel tumours.

One guideline (9 %) provided a definition. Ten guidelines (91 %) provided no definition of synchronous colorectal cancer and liver metastases.

*ii) Pre-operative staging and work-up.*

Six (55 %) guidelines recommended ^18^FDG-PET and liver MR for pre-operative staging. All were recommendations supported by low-quality evidence.

*iii) Treatment.*

Neoadjuvant chemotherapy was recommended as first treatment by 8 (73 %) of guidelines (Table 3). Two of these 8 guidelines [12,23] provided strong recommendations with high-quality evidence for this recommendation. The Japanese guidelines did not recommend this approach for patients with resectable disease.

Synchronous resection of liver metastases and primary tumour was supported by 7 (64 %) guidelines. One guideline (9 %) recommended against synchronous resection of liver metastases and primary tumour [15] and three guidelines made no recommendation either for or against synchronous hepatic resection and colectomy. Information on criteria for selection of patients with liver metastases for synchronous surgery was provided by three (27 %) guidelines – all with low or very low-quality evidence.

### Guidance on specific scenarios: pulmonary metastasectomy; synchronous resection after complete response

Synchronous pulmonary metastasectomy was supported by four (36 %) guidelines but all provided only low or very low-quality evidence for this approach. Synchronous surgery (liver and bowel surgery) after a complete response to neoadjuvant chemotherapy was discussed by two guidelines.

## Conclusions

This study has examined the advice provided by national and international guidelines for the management of patients with colorectal cancer and synchronous liver metastases. It is accepted that not all the information available to clinical teams will accrue from guidelines. For example, the EGOSLIM consensus specifically addresses the management of patients with colorectal cancer and synchronous liver metastases [2]. In addition, studies such as METASYNC and CoSMIC provide prospectively-collected, individual-patient data on the management of patients with colorectal cancer and synchronous liver metastases [24,25].

Relevant limitations of this study include the restrictions applied to the search criteria which reduced the number of articles eligible for the study, exclusion of reports not written in English and selection bias introduced by seeking guidelines reporting specifically on the management of patients with colorectal cancer and synchronous liver metastases.

What then can be learnt from this report? First, in terms of the quality of the guidelines, the AGREE II table highlights variation between guidelines. In addition, there are low overall scores for stakeholder involvement, rigour of development and editorial independence. Incorporation of patient or user views is important as these may differ from clinician recommendations [26]. Overall, variation in the quality of guidelines can lead to heterogeneity in recommendations.

Next, the specific advice provided by the guidelines on aspects of care was reviewed. Given the evidence of variation in definition of the term “synchronous” liver metastases [27] the fact that only one guideline provided a definition could introduce heterogeneity. Six (55 %) recommend ^18^FDG-PET and liver MR as part of the work-up for liver surgery although none cite high-quality evidence for these recommendations. Equally, it is noteworthy that 5 (45 %) guidelines recommend neither of these tests. Considering the differential sensitivity between CT and MR this difference is likely to contribute to difference in management and outcome [28].

In terms of treatment, neoadjuvant chemotherapy was advocated by 8 (73 %) with high-quality evidence being cited by two [12,23]. In relation to sequence of treatment, the bowel-first route was advocated by six guidelines and the liver-first route by seven. Synchronous liver and bowel surgery was recommended by seven (64 %) and three guidelines made no recommendation. One guideline recommended against synchronous surgery for synchronous disease. These approaches are not mutually exclusive. For example, five guidelines recommended all three approaches. For patients in whom there was a complete radiological response of the liver metastatic disease to neoadjuvant chemotherapy, hepatic resection was weakly recommended by only two guidelines [20,21] based on very low-quality evidence. The management of liver metastases which cease to be detectable on cross-sectional imaging by MR after systemic chemotherapy is clearly an area for further investigation.

For clinicians seeking to select patients for synchronous resection of liver and bowel tumours, the information provided by guidelines is limited.

In summary, this study is thought to be the first to overview guidelines on the management of patients with colorectal cancer and synchronous liver metastases. The guidelines allow for substantial variation in management reflecting the limited data to guide decision. The reasons for these variations could be due to differences in sampling of the literature, differential citing of evidence or reflections of differences in national practice.

The key recommendation of this study is that the available evidence could be better used by more collaboration between societies both nationally and internationally. Guidelines should provide clear definitions of the terms used and rather than be “stand-alone” documents there is a responsibility to seek consensus in recommendation across professional societies accepting that from global perspective healthcare systems have different financial constraints.

## Statement of ethics

An ethics statement is not applicable because this study is based exclusively on published literature.

## Funding sources

AKCC was supported by a non-restricted educational grant from the Dickinson Trust, Manchester Royal Infirmary.

## Other disclosures

No generative artificial intelligence (AI) and AI-assisted technologies were used in the study or the writing or preparation of the manuscript.

## CRediT authorship contribution statement

**Anthony K.C. Chan:** Writing – review & editing, Writing – original draft, Validation, Investigation, Formal analysis, Data curation. **Ajith K. Siriwardena:** Writing – review & editing, Writing – original draft, Visualization, Methodology, Formal analysis, Conceptualization.

## Declaration of competing interest

None.
